# Consuming royal jelly alters several phenotypes associated with overwintering dormancy in mosquitoes

**DOI:** 10.3389/finsc.2024.1358619

**Published:** 2024-06-07

**Authors:** Olivia E. Bianco, Aisha Abdi, Matthias S. Klein, Xueyan Wei, Cheolho Sim, Megan E. Meuti

**Affiliations:** ^1^ Department of Entomology, The Ohio State University, Columbus, OH, United States; ^2^ Department of Animal Science, McGill University, Ste. Anne de Bellevue, QC, Canada; ^3^ Department of Biology, Baylor University, Waco, TX, United States

**Keywords:** reproductive diapause, major royal jelly protein 1 (MRJP1), metabolomics, NMR spectroscopy, machine learning, qRT-PCR, RNA interference (RNAi)

## Abstract

**Introduction:**

Females of the Northern house mosquito, *Culex pipiens*, enter an overwintering dormancy, or diapause, in response to short day lengths and low environmental temperatures that is characterized by small egg follicles and high starvation resistance. During diapause, *Culex pipiens* Major Royal Jelly Protein 1 ortholog (CpMRJP1) is upregulated in females of *Cx. pipiens*. This protein is highly abundant in royal jelly, a substance produced by honey bees (*Apis mellifera*), that is fed to future queens throughout larval development and induces the queen phenotype (e.g., high reproductive activity and longer lifespan). However, the role of CpMRJP1 in *Cx. pipiens* is unknown.

**Methods:**

We first conducted a phylogenetic analysis to determine how the sequence of CpMRJP1 compares with other species. We then investigated how supplementing the diets of both diapausing and nondiapausing females of *Cx. pipiens* with royal jelly affects egg follicle length, fat content, protein content, starvation resistance, and metabolic profile.

**Results:**

We found that feeding royal jelly to females reared in long-day, diapause-averting conditions significantly reduced the egg follicle lengths and switched their metabolic profiles to be similar to diapausing females. In contrast, feeding royal jelly to females reared in short-day, diapause-inducing conditions significantly reduced lifespan and switched their metabolic profile to be similar nondiapausing mosquitoes. Moreover, RNAi directed against *CpMRJPI* significantly increased egg follicle length of short-day reared females, suggesting that these females averted diapause.

**Discussion:**

Taken together, our data show that consuming royal jelly reverses several key seasonal phenotypes of *Cx. pipiens* and that these responses are likely mediated in part by CpMRJP1.

## Graphical Abstract

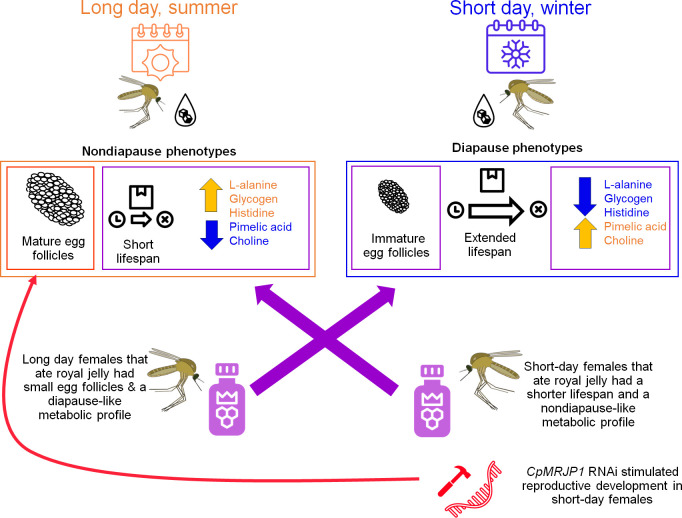

## 1 Introduction

Females of the Northern house mosquito, *Culex pipiens*, enter diapause in response to short daylengths and low environmental temperatures that act as harbingers of the approaching winter (1). Diapause allows mosquitoes to survive unfavorable winter conditions (2, 3), and it involves a unique suite of behavioral, hormonal and metabolic changes (4–6). In *Cx. pipiens*, exposure to short days as eggs, larvae, pupae and early adults causes adult females to enter an adult reproductive diapause (7). Diapause in females of *Cx. pipiens* is characterized by reproductive arrest, resulting in a decrease in the size of egg follicles as females divert energy away from reproduction (8). Adult mosquitoes that enter diapause feed on sugar-rich plant nectar, causing an increase in whole-body fat content (5, 9). Several genes that regulate metabolism are differentially expressed between diapausing and nondiapausing female mosquitoes; specifically, Robich and Denlinger (5) found that a gene associated with lipid accumulation, *fatty acid synthase*, was upregulated in diapausing females of *Cx. pipiens*, while two genes that encode enzymes related to digesting a blood meal*, trypsin* and *chymotrypsin-like* proteins, were down-regulated. Although many of the genes involved in generating diapause phenotypes have been well-characterized, it is still unclear what genes and proteins regulate diapause and initiate largescale metabolic and behavioral changes. However, insulin signaling and the Forkhead Transcription Factor, FOXO, have been implicated in regulating diapause responses in *Cx. pipiens* and several other insect species [reviewed in (10, 11)].

Royal jelly is produced by worker honey bees (*Apis mellifera*), and is a rich source of amino acids, lipids, vitamins, and other nutrients (12). Specifically, Nagai and Inoue (13) found that royal jelly consists of water (50–60%), proteins (18%), carbohydrates (15%), lipids (3–6%) as well as smaller amounts of water-soluble vitamin B and related components including vitamin B_1_, vitamin B_2_, vitamin B_6_, biotin, acetylcholine, pantothenic acid, inositol, and nicotinic acid. Worker bees and drones feed on royal jelly for the first 3 and 5 days of larval life respectively, after which they feed on beebread (a type of fermented pollen) and honey. However, future queen bees consume royal jelly exclusively, and this induces female larvae to follow the queen developmental trajectory that is characterized by high reproductive outputs and a longer lifespan (14, 15). One protein in royal jelly, referred to as Major Royal Jelly Protein 1 (AmMRJP1), produces strong antibacterial activity and is the most abundant glycoprotein within royal jelly, constituting over 50% of total protein content (16, 17). Surprisingly, the gene encoding the ortholog of AmMRJP1 in *Cx. pipiens* (*CpMRJP1*) is upregulated by FOXO and is more abundantly expressed in whole bodies of diapausing females of *Cx. pipiens* (18). The tissue specificity of *CpMRJP1* transcription and translation as well as the function of CpMRJP1 in diapausing mosquitoes has not been characterized. However, the upregulation of *CpMRJP1* in diapausing mosquitoes is surprising because these females are not reproductively active but do live substantially longer than nondiapausing females, especially in the absence of food (8).

Although it is unclear how increasing the abundance of AmMRJP1 or its orthologs impacts animals, several studies demonstrate that consuming royal jelly affects the physiology of invertebrates and mammals, including humans. Integrating royal jelly into human diets can improve reproductive health, combat neurodegenerative disorders, slow aging, and promote wound-healing (19). In rats, proteins in royal jelly can also function as antioxidants, protecting the testes of males against oxidative stress (20). Additionally, supplementing the diets of male rams with royal jelly increases sperm motility and viability (21). Similarly, royal jelly positively impacts fertility as well as semen quality and quantity in male rabbits (22). Introducing royal jelly into the diet of *Drosophila melanogaster* (L.) extends adult lifespan in males and females, possibly by increasing antioxidant activity, and stimulates feeding behavior and fecundity in females (23). Royal jelly also extends adult lifespan in the nematode *Caenorhabditis elegans* (Maupas) (24), suggesting that royal jelly may promote longevity across a wide range of invertebrates. Moreover, Fischman et al. (25) demonstrate that consuming royal jelly enhances the likelihood that alfalfa leaf cutting bees will enter diapause. While several studies have examined the role of royal jelly in animals, little is known about how consuming royal jelly might influence the seasonal responses and metabolic profile of mosquitoes, and to what extent these changes are induced by MRJP1.

Although metabolomic studies to uncover the differences between diapausing and nondiapausing *Cx. pipiens* have not been completed, previous research has examined metabolic differences between diapasuing and nondiapausing Asian tiger mosquitoes, *Aedes albopictus* (Skuse), flesh flies *Sarcophaga crassipalpis* (Macquart), and parasitic wasps, *Nasonia vitripennis* (Walker) (26–28). Not surprisingly, metabolomic studies demonstrate that diapausing mosquitoes, flesh flies, and parasitic wasps upregulate metabolites that act as cryoprotectants (26–28). In diapausing *N. vitripennis* the abundance of members of the glycolysis pathway are more abundant, reflecting an overall perturbation of the metabolic pathways in diapause (28). In *Ae. albopictus*, the monoamine neurohormones dopamine and octopamine, as well as phosphocholine and oleoyl glycine were more abundant in nondiapausing eggs compared to diapausing eggs (26). One objective of this study is to identify specific metabolites that are differentially abundant between diapausing and nondiapausing *Cx. pipiens* and how consuming diets that include royal jelly influences the overall metabolome of long and short-day reared mosquitoes.

Females of *Cx. pipiens* (L.) transmit pathogens that cause St. Louis encephalitis (29), West Nile virus (30), and canine heartworm (31) that infect millions of humans and animals each year (32). Female mosquitoes transmit these pathogens when they take a bloodmeal from a human or animal host (33). However, disease transmission is not equally distributed across time (34), in large part because diapausing mosquitoes no longer ingest blood (6) and, as a result, no longer transmit diseases (34). Therefore, uncovering molecular regulators of diapause in mosquitoes and other blood-feeding arthropods may offer novel opportunities to control these disease vectors and thereby improve human and animal health.

We first conducted phylogenetic analyses to determine how the sequence of *CpMRJP1* compared to that in other insects. To characterize how consuming royal jelly affects seasonal phenotypes in mosquitoes, we measured the abundance of *CpMRJP1* mRNA transcripts, reproductive development, lifespan, overall fat and protein content, as well as the metabolic profile of long and short-day reared females of *Cx. pipiens* that had consumed sugar water only (control) and those that consumed diets that included royal jelly. We also assessed which, if any, of the physiological impacts of royal jelly on seasonal responses were mediated by CpMRJP1 by using RNA interference (RNAi) to knock down this transcript in mosquitoes reared in long-day, diapause-averting and short day, diapause-inducing conditions. We hypothesized that mosquitoes that consumed diets including royal jelly would enter a diapause-like state regardless of environmental conditions, characterized by small egg follicles and increased longevity. Accordingly, we hypothesized that long and short-day reared mosquitoes that consumed royal jelly, and therefore had higher levels of AmMRJP1 within their guts, would exhibit a metabolic profile that was similar to that of diapausing mosquitoes that consumed sugar water. In contrast, we hypothesized that knocking down *CpMRJP1* with RNAi would prevent mosquitoes reared in short-day, diapause-inducing conditions from entering diapause and would decrease longevity.

## 2 Materials and methods

### 2.1 Sequence alignment and phylogenetic analyses

The amino acid sequence of *Culex quinquefasciatus* MRJP1 (CPIJ008700) was extracted from Vectorbase (https://vectorbase.org/vectorbase/app/) and fed into NCBI blastp search (https://blast.ncbi.nlm.nih.gov/Blast.cgi) to identify the potential evolutionary origin of this protein. Amino acid sequences from the top dipteran matches were extracted and used for comparison and determination of phylogenetic relationships among dipteran species. As Drapeau et al. (14) indicate that *MRJP1* is closely-related to the *yellow* gene in insects, the amino acid sequences of *yellow* from *Culex quinquefasiatus*, extracted from Vectorbase, and *yellow* and its associated paralogs in *Drosophila melanogaster*, extracted from FlyBase, were used for the phylogenetic analysis. The following amino acid sequences were used to make the phylogenetic tree: *MRJP1* from *Cx. quinquefasciatus* (XP_001850268.2), *MRJP1* from *Apis mellifera* (NP_001011579.1), *MRJP1* from *Aedes aegypti* (XP_021693794.1), *MRJP1-like* from *Sabethes cyaneus* (XP_053682299.1), *MRJP1-like isoform X2* from *Anopheles funestus* (XP_049285312.1), *yellow* from *Cx. quinquefasiatus* (CQUJHB005397), *yellow* from *D. melanogaster* (FBgn0004034), *yellow-like* from *Ae. albopictus* (XP_019534039.2), *yellow-like* from *Anopheles maculipalpis* (XP_050069861.1), *yellow-like* from *An. merus* (XP_041787038.1), *yellow-like* from *An. nili* (XP_053679995.1), *yellow-like isoform X1* from *An. funestus* (XP_049285311.1), *yellow-like* from *An. moucheti* (XP_052891110.1), *yellow-like* from *An. marshallii* (XP_053669181.1), *yellow-like* from *An. stephensi* (XP_035901855.1), *yellow-like* from *An. cruzii* (XP_052868860.1), *yellow-like* from *An. aquasalis* (XP_050087457.1). The following paralogs from *D. melanogaster* were also included: *yellow-b* (FBgn0032601), *yellow-c* (FBgn0041713), *yellow-d* (FBgn0041712), *yellow-d2* (FBgn0034856), *yellow-e* (FBgn0041711), *yellow-e2* (FBgn0038151), *yellow-e3* (FBgn0038150), *yellow-f* (FBgn0041710), *yellow-f2* (FBgn0038105), *yellow-g* (FBgn0041709), *yellow-g2* (FBgn0035328), *yellow-h* (FBgn0039896).

Sequence alignment was performed using Clustal Omega (https://www.ebi.ac.uk/jdispatcher/msa/clustalo) and phylogenetic analyses were conducted using the maximum likelihood method in MEGA 11 software, with bootstrap values calculated from 500 trees (35). To characterize the protein composition and domains, we conducted an InterProScan search (https://www.ebi.ac.uk/interpro/search/sequence/).

### 2.2 Mosquito rearing

A colony of *Culex pipiens* established in June 2013 from Columbus, Ohio (Buckeye strain) was used in this experiment. From the time that they were first instar larvae and until the adults were collected for analyses, mosquitoes were reared at 18°C and exposed to either long-day, diapause-averting conditions (photoperiod of Light: Dark 16:8 hr) or short-day, diapause-inducing conditions (photoperiod of L:D 8:16 hr). Larvae were reared in plastic containers filled with reverse osmosis water and were fed a diet of ground fish food (Tetramin Tropical Fish Flakes) according to the procedure described by Meuti et al. (36). To optimize our metabolomic procedure and allow us to acquire spectra from single mosquitoes, we conducted a preliminary experiment where mosquito pupae were transferred to cages that contained sugar water only. For all subsequent experiments, pupae from both the long and short-day photoperiods were randomly and equally divided into two cages, one that contained sugar water only (control) and one that contained 2 g of Royal Jelly (Starkish) that was dissolved in 1.5 mL of 10% sucrose solution (experimental treatment). Adult mosquitoes consumed their prescribed dietary treatments *ad libitum* (4 treatments total; n≅150 adults/treatment). One week after peak adult emergence, mosquitoes were euthanized and collected for experimental analyses.

### 2.3 Measuring the abundance of *MRJP1* mRNA

Quantitative real time PCR (qRT-PCR) was used to assess how the abundance of *CpMRJP1* was affected by supplementing the diet of adult mosquitoes with royal jelly. The procedure for qRT-PCR was based on (37). We designed primers for *CpMRJP1* (CPIJ008700-RA) using Primer3 (Forward: TGAACGATCGTCTGCTGTTC; Reverse: TCCTCCCACATGGTATCGTT; (38). A standard curve verified that the primers met the MIQE guidelines (39). RNA was isolated from female mosquitoes (n=5 females/biological replicate; 5 biological replicates/rearing condition and feeding treatment) one week following adult emergence using TRIzol according to the manufacturer’s instructions. Complementary DNA (cDNA) was synthesized using the SuperScript III kit (Invitrogen), following the manufacturer’s instructions. All qRT-PCR reactions were done in triplicate on a 96-well plate using a CFX Connect qRT-PCR machine (BioRad). Each well contained a 10 μL reaction, consisting of 5 μL of iTaq Universal SybrGreen Supermix (BioRad), 400 nM of forward and reverse primers for either *CpMRJP1* or our reference gene (*Ribosomal Protein 19; RpL19;* 39), 3.2 μL of molecular grade water, and 1 μL of cDNA. qRT-PCR reactions were completed through an initial denaturation at 94°C for 2 min, followed by 40 cycles at 94°C for 15 sec and 60°C for 1 min. A melt curve was run after each qRT-PCR reaction to ensure that only a single PCR product was produced. The abundance of *CpMRJP1* transcripts was normalized to the abundance of *RpL19* using the 2^-ΔCT^ method as previously described (40). We ran a model to ensure that the abundance of *RpL19* mRNA did not differ significantly among dietary treatments (p = 0.778) and therefore was a suitable reference gene for this study.

### 2.4 Assessing diapause status

To assess the diapause status of long and short day-reared females that had consumed sugar water (control) or diets that included royal jelly, we used two common markers of diapause: egg follicle length and fat content. One-week after adult emergence, we randomly euthanized 20 female mosquitoes from each dietary and rearing treatment and dissected their ovaries and egg follicles in a 0.9% saline solution (NaCl). The length of 10 egg follicles/female were measured at 200 times magnification using an inverted microscope (Nikon; n = 20 females/treatment). We also randomly selected eight, one-week-old females from the same cohorts and dietary treatments and measured the fat content in each female mosquito using a Vanillin lipid assay (41) that was modified to allow us to measure samples using a plate reader (42). The data were normalized by dividing the measured lipid content by the lean mass of the whole-body mosquito (lean mass = μg mosquito wet mass - μg of lipid).

### 2.5 Measuring protein content

As royal jelly is protein-rich, we also wanted to determine whether supplementing the diet with royal jelly affected the whole-body protein content of female mosquitoes. The protein content within eight, randomly selected individual female mosquitoes from the same cohort and dietary treatments as the lipid and egg follicle treatments was measured using a Bradford Assay kit (BioRad) following the manufacturer’s instructions (43). In brief, each female mosquito was weighed and then homogenized in 200 μL of a 10% ethanol solution. Samples were added in triplicate to a 96-well plate, and 250 μL of Quick Start Bradford 1X Dye Reagent (BioRad) was added to each well. The absorbance of each sample was measured using a FLUOStar Omega Microplate Reader. Measurements were normalized by dividing the protein content by the wet mass of each female mosquito (44).

### 2.6 Evaluating longevity in the absence of food

In the field, diapausing females are able to survive for 3–6 months in without access to food (45). Therefore, we wanted to determine how dietary conditions affected the lifespan of female mosquitoes in the absence of food. Long and short-day reared mosquito pupae were placed in cages with access to sugar water or royal jelly (4 treatments total). One week after peak adult emergence, all male mosquitoes as well as the royal jelly or sugar-water food sources were removed from each cage. The remaining females (n = 70 – 75 females/treatment) were counted and allowed constant access to water. Every week thereafter we counted and removed dead females from the cage until no female mosquitoes remained.

### 2.7 Performing metabolomic analyses

The experimental protocol for all metabolomic analyses employed tissue extraction followed by Nuclear Magnetic Resonance (NMR) analysis and was based on the procedure described in Wu et al. (46). One mosquito was weighed and placed in a 2 mL microtube with approximately 750 mg of ceramic beads. For our initial experiment, 3 short-day reared, diapausing and 3 long-day reared, nondiapausing mosquitoes that had consumed sugar water only were used. For our second experiment, 10 independent female mosquitoes from each photoperiodic and dietary treatment, and from the same cohort as the egg follicle, fat content, and protein measurement experiments were randomly selected (n = 40 total). Mosquito samples were homogenized in 400 µL cold methanol and 85 µL water, and the homogenate was transferred to a separate tube without beads. Next, 400 µL chloroform and 200 µL water were added to the homogenate, which was then vortexed and centrifuged (2,000 rcf for 5 min at 4°C). The aqueous (methanol) layer was isolated and collected in a new 1.5 mL microtube before being dried in an evaporator. Deuterium oxide (heavy water), trimethylsilylpropanoic acid (TSP), and boric acid were added to the evaporated extracts and vortexed. The pH of the samples was manually adjusted to 7.4 and then transferred to 5 mm NMR tubes.

The metabolites in each mosquito sample were measured using NMR spectroscopy following the procedure detailed in (47). 1D ^1^H NOESY spectra were obtained for the aqueous extracts. In addition, one ^1^H-^13^C HSQC spectrum of a pooled sample was acquired for the four separate dietary and photoperiodic conditions according to (48). Avance III HD 800 and Ascend 850 MHz spectrometers, each with an inverse cryoprobe and z-gradients (Bruker BioSpin, Billerica, MA), were utilized to obtain NMR measurements, and resulting NMR spectra were analyzed as described in Newell et al. (47) and (49). Topspin 3.6.1 and AMIX 3.9.15 software (Bruker BioSpin, Billerica, MA) were used for preprocessing. The untargeted approach of spectral binning was chosen, where each spectrum is divided into small sections, or bins, which are subsequently analyzed. It should be noted that this approach provides broad coverage across various classes of metabolites, as compared to a targeted approach where only a predefined subset of metabolites of interest is quantified. In the initial experiment to optimize the protocol, 1D NMR spectra were binned with a bin width of 0.005 ppm. In contrast, for the second experiment that evaluated the effects of consuming diets that included royal jelly, the bin width was lowered to 0.003 ppm because these measurements were taken using a higher-frequency instrument (850MHz). Spectra were binned using the R package mrbin [Version 1.5.0; (50)}. Signals that showed large inter-spectra chemical shift differences were manually added to broader bins. Noise signals were automatically removed, and data was scaled using PQN (probabilistic quotient normalization) to correct for differences in sample mass and extraction efficacies. Each bin was then scaled to unit variance. Principal Component Analysis (PCA) models were generated to visualize metabolic data and screen for outliers regarding data quality. Signals of interest were identified using public databases and identifications were validated using the acquired HSQC spectrum and measurements of pure samples.

### 2.8 Assessing the effect of knocking down *MRP1* with RNAi

RNA interference (RNAi) was used to knock down *CpMRJP1* mRNA to evaluate how this protein affects the diapause status of female mosquitoes. The procedure for RNA interference was based on the protocol detailed in (37). Double-stranded RNA (dsRNA) specific to *CpMRJP1* and *Beta-galactosidase* from *E. coli* (*β-gal*; control) were synthesized using the Promega T7 RNAi Express Kit according to the manufacturer’s instructions. We selected *β-gal* dsRNA as a control because mosquitoes do not consume lactose and hence genes encoding *β-gal* proteins are absent from their genomes, and because we have previously used *β-gal* dsRNA as a control in RNAi experiments (37, 40). We designed primers to synthesize a 230 bp fragment of *CpMRJP1* (CPIJ008700-RA) in *Cx. pipiens* using Primer3 (Forward: CACCGCCAAACCGAACAAAT; Reverse: TGAGCAGCCCAAAGTACAGG; 37), which served as the template to create dsRNA. On the day of adult emergence, 3 μg of either *β-gal* or *CpMRJP1* dsRNA was injected into the thorax of long and short day-reared mosquitoes. Following injection, females were placed into small plastic containers (4.62 x 6.75 x 7.19 inches) where they consumed 10% sucrose solution *ad libitium*. To confirm gene knockdown, RNA was isolated using Trizol according to the manufacturer’s instructions from 3 biological replicates each containing 5 whole-body, female mosquitoes that were euthanized two days after dsRNA injection. cDNA was synthesized and qRT-PCR was conducted as described above (section 2.3), except that we used *CpMRJP1* qRT-PCR primers that were previously designed by Sim et al. (18), and that after normalizing *CpMRJP1* expression to the *RpL19* reference gene, *CpMRJP1* expression was again normalized to its expression in *β-gal*-dsRNA injected mosquitoes from the same photoperiodic condition (40).

To determine how *CpMRJP1* dsRNA affected seasonal phenotypes, the egg follicle lengths (n = 20) and fat content (n = 8) of randomly selected females were measured ten days following dsRNA injection as described above (section 2.4). After feeding on sugar water for one week, the lifespan of *β-gal* or *dsMRJP1* dsRNA-injected females (n = 37 – 49 females/treatment) in the absence of food was also measured as described above (section 2.5).

### 2.9 Data analysis

All data analyses were conducted in R version 3.3.3 (51). Two-way ANOVAs and Tukey’s *post-hoc* tests were used to determine whether dietary treatment and/or photoperiod significantly affected *CpMRJP1* mRNA abundance, egg follicle length, lipid content, and protein content in female mosquitoes. Student’s T-tests were used to determine whether injecting *CpMRJP1* dsRNA effectively reduced *CpMRJP1* mRNA abundance, while two-way ANOVAs were used to determine whether dsRNA injection and photoperiod significantly affected the egg follicle length or fat content. A value of alpha < 0.05 was applied to discern statistical significance.

To determine how supplementing the diet with royal jelly and knocking down *CpMRJP1* with RNAi affected the longevity of female mosquitoes in the absence of food, we used Cox-proportional hazards models (survival package) that included the effects of diet and photoperiod. Hazard ratios were obtained from these models as an estimate of the ratio between the risk of dying between photoperiodic and dietary treatments, where negative hazard ratios (HR) indicate a protective effect. Kaplan Meier survival curves were also plotted and used to provide the median survival time or the time at which 50% of the population was still alive as defined by (52, 53), and log-rank tests were used to determine significant differences between treatments.

For analysis of NMR metabolomics data, for each spectral bin, a general linear model (GLM) was created to account for the effect of diet (sugar water or royal jelly), photoperiod (long or short day-rearing conditions), and the interaction term between diet and photoperiod. To correct resulting p-values multiple testing, we used a False Discovery Rate (FDR) of 5% (54). Signals that were significant in the GLM analysis were tested for pairwise group differences using Tukey’s Honest Significant Difference (HSD) test. An Artificial Neural Network (ANN) was generated using R packages keras (version 2.11.1) and tensorflow (version 2.11.0). A dense network was generated with one input neuron per NMR bin (N=1823), 200 hidden neurons with ReLU activation function, and 2 output neurons, representing “short day” and “long day” rearing conditions. The ANN was trained on the data from the “sugar water” group using leave-one-out cross validation. Samples from the “royal jelly” group were then predicted using the trained ANN.

Pathway enrichment analysis was performed by downloading KEGG pathway maps of *Culex pipiens pallens* with KEGG code cpii (55) using the R package KEGGREST (version 1.36.3). Fisher’s Exact Test was used to assess the number of matching metabolites per pathway. A pathway uniqueness score *u* was calculated as follows: For each pathway, each observed metabolite was assigned the inverse of the number of pathways in which this metabolite occurs organism-wide, then the maximum value was chosen as the pathway uniqueness score. Therefore, higher values of *u* indicate pathways with more metabolites that uniquely occur in that respective pathway, and we further investigated pathways with *u*≥0.2.

## 3 Results

### 3.1 Phylogenetic analysis of *Culex pipiens MRJP1*

**Figure 1:**
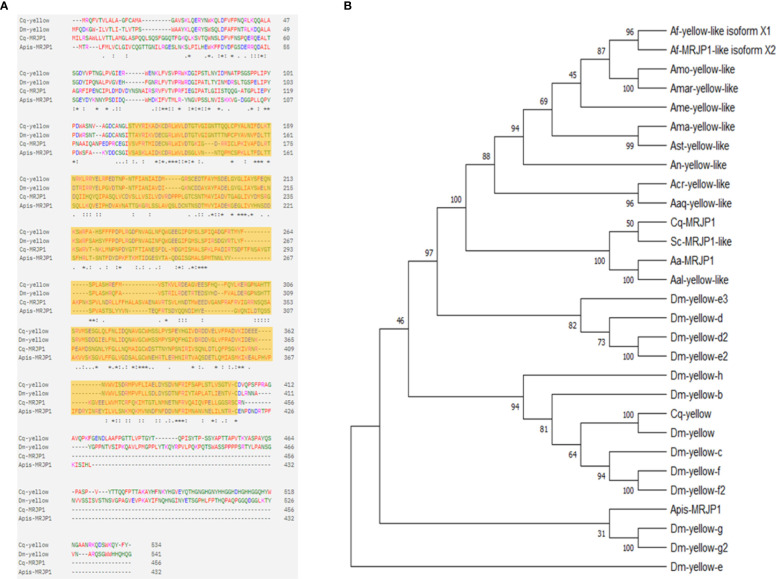
Phylogenetic analysis and sequence alignment of MRJP/Yellow family proteins’ sequences in insect species. **(A)** Sequence alignment on the amino acid sequences of Yellow and MRJP1 proteins from *Culex quinquefasciatus*, *Drosophila melanogaster*, and *Apis mellifera*. The highlighted region indicates the conserved MRJP domain present in all four sequences; the . : or * indicate amino acid residues that are conserved across 2, 3 or 4 sequences. **(B)** The phylogenetic tree was generated using MEGA v.11 maximum likelihood estimation with bootstrap analysis. The bootstrap consensus tree inferred from 500 replicates is taken to represent the evolutionary history of the taxa analyzed (Felsenstein, 1985). The percentage of replicate trees in which the associated taxa clustered together in the bootstrap test 500 replicates are shown next to the branches. Apis, *Apis mellifera*; Aal, *Aedes albopictus*; Ast, *Anopheles stephensi;* An, *Anopheles nili*; Acr, *Anopheles cruzi*i; Aa, *Aedes aegypti*; Amo, *Anopheles moucheti*; Amar, *Anopheles marshallii*; Sc, *Sabethes cyaneus*; Aaq, *Anopheles aquasalis*; Af-yellow, *Anopheles funestus*; Ame, *Anopheles merus*; Cq, *Culex quinquefasciatus*; Ama, *Anopheles maculipalpis*; Af, *Anopheles funestus*; Dm, *Drosophila melanogaster*.

To identify the evolutionary relationships of *Cx. pipiens* MRJP1 with MRJP/yellow homologues in other insects, we conducted a phylogenetic analysis using amino acid sequences of MRJP/yellow proteins from dipteran species. First, we conducted a BLAST search using the protein sequence of *Cx. quinquefasciatus* MRJP1 (CPIJ008700) and extracted the amino acid sequences of the top hits from dipteran species. We also obtained the protein sequences of Yellow in *Cx. quinquefasciatus* and *D. melanogaster*, as well as 13 Yellow-like proteins in *D. melanogaster* from Vectorbase (56). Multiple sequence alignment was performed to compare sequence identity between species. The sequence identity between *Cx. quinquefasciatus* MRJP1 and Yellow from *D. melanogaster* was found to be 23.41%, while it was 21.39% with Yellow from *Cx. quinquefasciatus*. This result is consistent with the high identity of 20–30% shared between MRJP and Yellow paralogs in *D. melanogaster*, suggesting a common evolutionary origin (57). Additionally, we performed a sequence identity comparison between MRJP1 proteins from *Cx. quinquefasciatus* and *Apis mellifera* to gain insights into the evolutionary and functional aspects of this protein. Our findings unveil that MRJP1 in these species share 129 identical amino acid residues, corresponding to a percent identity of 26.88%. Through alignment of the amino acid sequences between Yellow and MRJP1 in *Cx. quinquefasciatus*, *D. melanogaster*, and *Apis mellifera*, we identified a consensus conserved MRJP domain in all four sequences, indicating the preservation of the MRJP domain throughout insect evolution (**Figure 1A**).

We also conducted a functional analysis of *Cx. quinquefasciatus* MRJP1 using InterProScan and found that it belongs to the Royal jelly/protein yellow family in the InterPro database and the MRJP family in the Pfam database. This result is consistent with previous findings indicating that all Yellow proteins have a conserved MRJP domain (58). Subsequently, we used these sequences to create a phylogenetic tree to discover evolutionary relationships (**Figure 1B**). The percentage of replicate trees in which the associated H3 clustered together in 500 bootstrap replicates is shown adjacent to the branches. The tree was drawn to scale, with branch lengths in the same units as those of the evolutionary distances used to infer the phylogenetic tree. In the resulting phylogenetic tree, Yellow proteins in *Cx. quinquefasciatus* and *D. melanogaster* were found to be closely related and clustered together with all other Yellow paralogs in *D. melanogaster*. As expected, *Cx. quinquefasciatus* MRJP1 closely clustered with MRJP/Yellow-like proteins from other dipteran species identified in the BLAST top matches. However, MRJP1 was found to be farther away from Yellow from *Cx. quinquefasciatus* and *D. melanogaster* and other Yellow paralogs from *D. melanogaster* as well as MRJP1 in *A. meliferra* (**Figure 1B**). The distant relationship between Yellow of *Cx. quinquefasciatus* and *D. melanogaster* and MRJP1 in *Cx. quinquefasciatus* revealed by our phylogenetic analysis suggests that MRJP1 likely has different functions and targets than Yellow in *D. melanogaster*.

### 3.2 Measuring *MRJP1* mRNA abundance in response to rearing and dietary conditions

**Figure 2:**
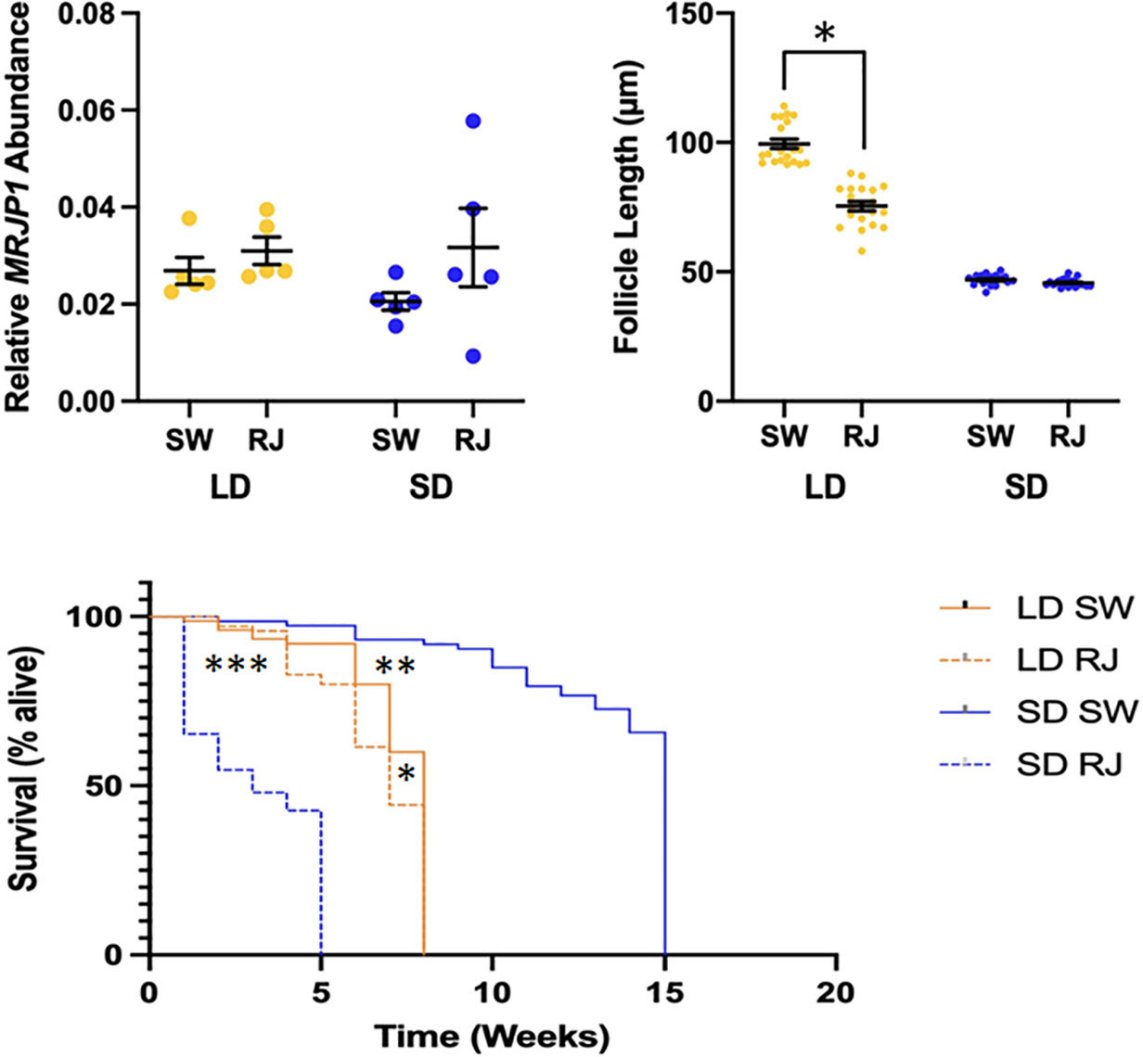
Phenotypic effects of consuming royal jelly (RJ) in mosquitoes. **(A)** Consuming RJ did not have any significant effect on relative *MRJP1* mRNA abundance in long day (LD) or short day (SD) reared mosquitoes. **(B)** Consuming RJ significantly decreases egg follicle length in long day (LD) mosquitoes. Significant difference denoted by * (Tukey’s *post-hoc* test; p < 0.001). **(C)** The lifespan of female mosquitoes was significantly different between long day (LD) and short day (SD) mosquitoes. The consumption of royal jelly (RJ) by mosquitoes in both rearing conditions led to a significant decrease in lifespan. Significant differences denoted by *** (SD RJ to SD SW; p < 0.001), ** (LD SW to SD SW; p < 0.001) and * (LD RJ to LD SW; p = 0.03).

The relative abundance of *MRJP1* mRNA did not change significantly in response to dietary treatment or photoperiodic conditions (**Figure 2A**). A two-way ANOVA revealed that there was not a statistically significant interaction between the effects of diet and photoperiod on the abundance of *CpMRJP1* mRNA (F_1,16_ = 0.589, p = 0.458). Simple main effects analysis showed that diet did not have a statistically significant effect on *CpMRJP1* abundance (F_1,16_ = 0.025, p = 0.876). Contrary to previous experiments (18), there was no significant difference in the abundance of *MRJP1* transcripts between females reared in long day or short-day conditions (F_1,16_ = 2.67, p = 0.122), likely due to high levels of variation in *CpMRJP1* transcript abundance in the short-day reared females.

### 3.3 Assessing the effects of royal jelly on mosquito diapause status and lifespan

Egg follicle length can be used to determine diapause status of female mosquitoes of *Cx. pipiens* (5,8), such that an average egg follicle length of less than 75 μm indicates diapause, follicle lengths between 75 and 90 μm indicates an intermediate state, and follicles greater than 90 μm indicates nondiapause (42). We used a two-way ANOVA to evaluate the effects of both photoperiod and diet on egg follicle size. Our model revealed that both photoperiod (F_1,76_ = 1008.2, p < 0.001) and diet (F_1,76_ = 95.32, p < 0.001) independently affected egg follicle length. Moreover, there was a significant interaction between photoperiod and diet on egg follicle length (F_1,76_ = 78.4; p < 0.001). As expected, one week after adult emergence all long-day reared females that consumed sugar water (controls) were in a clear nondiapause state (**Figure 2B**; average egg follicle length of 99.5 ± 1.8 μm). However, 50% of long-day reared females that consumed diets including royal jelly had egg follicles that were characteristic of being in diapause, while the remaining females were in an intermediate state, and none of the long day-reared females that had consumed diets including royal jelly had egg follicle lengths that were large enough to be considered in a nondiapause state (**Figure 2B**). Therefore, consuming royal jelly caused a significant decrease in the overall average egg follicle length in long-day reared females (average egg follicle length of 75.4 ± 1.8 μm; Tukey’s *post-hoc* test, p < 0.001, 95% CI = [19.3, 28.9]). Females reared in short-day conditions had significantly smaller egg follicles compared to those reared in long-day conditions (Tukey’s *post-hoc* test, p < 0.001, 95% CI = [-43.7, -38.5]). All females reared in short-day conditions were in a clear diapause state (**Figure 1B**), regardless of whether they consumed sugar water only (46.9 ± 0.4 μm) or diets that included royal jelly (45.7 ± 0.3 μm); therefore dietary treatment did not effect the egg follicle length of dietary treatment did not significantly impact egg follicle length of short day-reared females (Tukey’s *post-hoc* test, p = 0.92).

We also used a two-way ANOVA to assess whether dietary treatment and/or photoperiod affected the fat content of female mosquitoes (**Supplementary Figure S2A**), as previous studies demonstrate that diapausing females accumulate significantly higher levels of fat than nondiapausing mosquitoes (37, 42). Our analyses revealed that neither photoperiod (F_1,28_ = 0.378, p = 0.54) nor diet (F_1,28_ = 0.567, p = 0.458) affected fat content. However, there was a slight but non-significant interaction between photoperiod and diet (F_1,28_ = 3.91, p = 0.058). Short day-reared females that consumed sugar water had higher levels of lipid relative to those that consumed royal jelly, although this result was not statistically significant (SD RJ = 22.19 ± 2.46% lipid; SD SW = 29.48 ± 4.75% lipid; Tukey’s HSD, p = 0.822).

A two-way ANOVA of the effects of photoperiod and diet on protein content revealed that photoperiod (F_1,25_ = 4.97, p = 0.035) but not diet (F_1,25_ = 0.03, p = 0.865) significantly affected the protein levels within female mosquitoes. Our analyses also revealed that there was no significant interaction between photoperiod and diet on protein content (F_1,25_ = 0.03, p = 0.576). Females reared in long-day conditions had roughly the same amount of protein whether they consumed sugar water or diets that included royal jelly (**Supplementary Figure S2B**; average protein content LD RJ = 12.16 ± 0.76 μg/mg; LD SW = 12.60 ± 1.41 μg/mg; Tukey’s HSD, p = 0.99). Dietary treatment did not significantly affect the protein content of short-day reared females (SD RJ = 10.22 ± 0.98 μg/mg; SD SW = 9.33 ± 1.32 μg/mg; Tukey’s HSD; p = 0.95). However, females reared in short-day conditions contained significantly less protein than long-day reared females (Tukey’s HSD, p = 0.034, 95% CI = [-5.04, -0.200]).

As diapausing females can survive for prolonged periods without without food (reviewed in 11), we also assessed the starvation resistance of mosquitoes reared in different photoperiodic conditions that consumed either sugar water or diets including royal jelly for seven days prior to food removal (**Figure 2C**). Our Cox proportional hazards model revealed that photoperiod significantly impacted survival time in the absence of food, such that short-day reared females were significantly less likely to die relative to long-day reared females (**Supplementary Table S1**; z = -8.427; p < 0.001). Although consuming diets that include royal jelly did not significantly impact survival time relative to feeding on sugar water (**Supplementary Table S1**; z = 1.48; p = 0.138), there was a strong interaction between photoperiod and diet, such that under short-day conditions, consuming diets that included royal jelly significantly increased the risk of death (z = 12.3; p < 0.001). The median survival time of long-day reared females that consumed sugar water was eight weeks of age, while the median survival time of sugar-fed, short-day reared mosquitoes was 15 weeks (**Figure 2C**), and the risk of dying was significantly lower for short-day reared females (Tukey’s HSD, z = 8.47, p < 0.001). However, the opposite was observed with female mosquitoes that consumed diets including royal jelly; the median survival time of long-day reared mosquitoes that consumed royal jelly was 7 weeks while the median survival time of short-day reared, royal jelly-fed mosquitoes was 3 weeks, showing that under short-day conditions consuming diets that include royal jelly significantly increased the risk of dying (Tukey’s HSD, z = 10.58; p < 0.001). Under long-day conditions, consuming royal jelly did not affect the risk of dying (Tukey’s HSD, z = 1.482; p = 0.4486), whereas under short-day conditions, consuming diets that included royal jelly reduced the median survival time by 12 weeks and significantly increased the risk of death (Tukey’s HSD, z = 13.27; p < 0.001).

### 3.4 Characterizing the effects of royal jelly on the metabolomic profile of long and short day-reared mosquitoes

**Figure 3:**
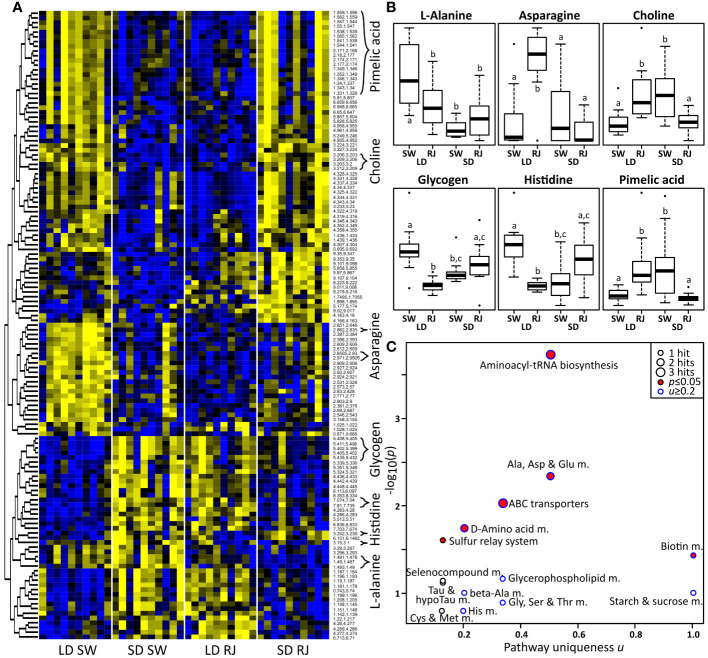
Consuming royal jelly reverses seasonal differences in the mosquito metabolome. **(A)** Heat map of NMR signals that were significantly different between treatment groups. Signals that were unambiguously identified are labeled with the respective metabolite name. Yellow represents metabolites that were highly abundant, while blue represents metabolites that were less abundant. **(B)** Boxplots of significantly altered metabolites (arbitrary units). Different superscript letters indicate differences with p ≤ 0.05. **(C)** Pathway enrichment analysis plot. RJ, royal jelly; SW, sugar water; SD, short-day, diapause-inducing conditions; LD, long-day, diapause-averting conditions; m., metabolism; Tau, taurine.

Preliminary data collected during initial optimization of the metabolomics protocol suggests that there are largescale differences in the metabolic profile of diapausing and nondiapausing *Cx. pipiens* (**Supplementary Figure S2A**). A Principal Component Analysis (PCA) reveals partial group separations for both the photoperiodic conditions and consuming royal jelly, indicating that both factors affect the metabolism of female mosquitoes (**Supplementary Figure S2B**). It should be noted that PCA does not search for differences between groups of interest, but it rather separates samples according to signals of largest variance. Frequently in metabolomics studies, confounding factors obscure PCA group separations by adding to the overall variance of the dataset. Therefore, additional analyses were performed to find differential signals. General linear models (GLM) revealed that 168 out of 1823 spectral signals significantly changed in response to at least one of the following: photoperiod, diet, or their interaction (**Supplementary Table S1**). **Figure 3A** shows a heatmap of all signals that significantly changed. Notably, nondiapausing mosquitoes that consumed sugar water exhibit strong metabolic differences compared to diapausing mosquitoes that also consumed sugar water (**Supplementary Table S1**; **Figure 3A**). Visual inspection revealed that mosquitoes reared under long-day, diapause-averting conditions switch to a “diapause-like” metabolic profile after consuming royal jelly. In contrast, females reared under short-day, diapause-inducing conditions switched to a “nondiapause-like” metabolic state after consuming royal jelly (**Supplementary Table S2**; **Figure 3A**). To confirm these qualitative observations, an artificial neural network (ANN) was trained on the sugar water group to predict long-day or short-day rearing conditions. Using leave-one out cross validation of each SW sample (**Supplementary Table S2**) reveals that the ANN correctly predicted most short-day (“diapause”) and long-day (“nondiapause”) samples in the sugar water group. However, when using the ANN to predict the photoperiod of mosquitoes that had consumed royal jelly (**Supplementary Table S3**), most short-day samples were predicted as being long-day, and most long-day samples were predicted to be short-day. The ANN results thus confirm that consuming royal jelly switches the metabolic profile of long-day reared females to be “diapause-like” and short-day reared females to be “nondiapause-like.”

Among the significant signals (**Supplementary Table S2**), six metabolites could be unambiguously identified using existing information in NMR databases (**Supplementary Table S3**; **Figure 3B**). Short day-reared, diapausing mosquitoes that consumed sugar water and long day-reared mosquitoes that consumed royal jelly had significantly higher levels of pimelic acid, asparagine, and choline; but significantly lower levels of L-alanine, histidine, and glycogen, compared to long-day mosquitoes that consumed sugar water (**Figure 3B**; see **Supplementary Table S3** for GLM coefficients and Tukey’s HSD p-values). Identical trends are seen in mosquitoes reared in short-day, diapause-inducing conditions that consumed sugar water (significant in all except asparagine). In contrast, short day-reared mosquitoes that consumed royal jelly showed opposite metabolic trends when compared to short-day, sugar-fed controls; however, this difference was only significant for choline and pimelic acid.

Metabolic pathway enrichment results indicate that several pathways were affected by photoperiod and diet. When plotting the negative decadic logarithm of the p-value versus the uniqueness score *u*, pathways of interest are expected to be found toward the top (p ≤ 0.05) or the right (*u* ≥ 0.2) in the resulting scatterplot (**Figure 3C**). Pathways of high interest were found to be Alanine, Aspartate and Glutamate Metabolism (p = 0.0044, *u* = 0.5), Biotin Metabolism (p = 0.036, *u* = 0.5), Starch and Sucrose metabolism (*u* = 1.0), Glycerohospholipid metabolism (*u* = 0.33), and Histidine Metabolism (*u* = 0.33). Some pathways such as Aminoacyl-tRNA biosynthesis, ABC transporters, Sulfur relay system, and D-amino acid metabolism were significant. However, these significant differences were caused by repetitive metabolic reactions in these pathways and/or non-specific reactions, therefore we chose to treat these results as statistical artifacts and do not further discuss them.

### 3.5 Effects of *CpMRJP1* dsRNA on mosquito diapause status and survival

**Figure 4:**
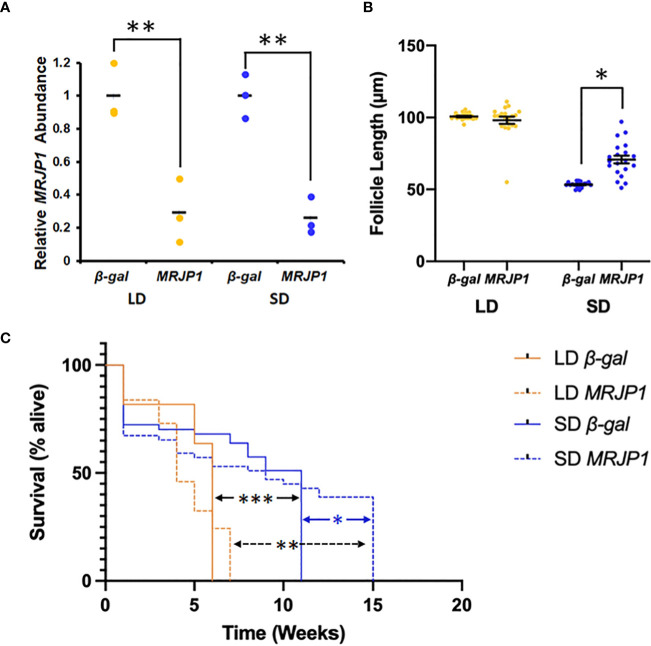
dsRNA against *MRJP1* affects seasonal phenotypes in mosquitoes. **(A)** Treatment with dsRNA for *MRJP1* significantly reduced the abundance of relative *CpMRJP1* mRNA abundance relative to *β-gal* dsRNA-injected controls in long day (LD) or short day (SD) mosquitoes. Significant difference denoted by * (p < 0.01). **(B)** dsRNA against *MRJP1* caused a significant increase in egg follicle length in short day (SD) mosquitoes. Significant difference denoted by * (p < 0.001). **(C)** dsRNA against *MRJP1* significantly affected the lifespan of (SD) conditions. Significant differences denoted by *** (LD *β-gal* to SD *β-gal*; p < 0.001), ** (LD *MRJP1* to SD *MRJP1*; p < 0.001) and * (SD *β-gal* to SD *MRJP1*; p = 0.0187).

A knockdown confirmation analysis was performed to determine if *CpMRJP1* dsRNA significantly reduced the abundance of *CpMRJP1* mRNA transcripts (**Figure 4A**). We found that *MRJP1* dsRNA significantly reduced the abundance of *CpMRJP1* transcripts in both long-day (71% reduction in transcript abundance relative to *β-gal* dsRNA-injected controls; T = 4.75; p = 0.009) and short-day reared females (74% reduction in transcript abundance relative to *β-gal* dsRNA-injected controls; T = 7.40, p = 0.0018).

Knocking down *CpMRJP1* dsRNA significantly affected egg follicle length in short-day reared females (**Figure 4B**). A two-way ANOVA was used to determine how photoperiod and dsRNA injection affected egg follicle length, and revealed that both photoperiod (F_1, 76_ = 394; p < 0.001) and dsRNA treatment (F_1, 76_ = 15.4; p = 0.0019) had significant effects. Moreover, there was a significant interaction between photoperiod and dsRNA treatment (F_1, 76_ = 28.3; p <0.001). All females reared in long-day conditions that were injected *β-gal* and *CpMRJP1* dsRNA were in a clear nondiapause state, such that the average egg follicle length of *β-gal* dsRNA-injected (100.7 ± 0.5 μm) and *CpMRJPI* dsRNA-injected mosquitoes (98.1 ± 2.5 μm) were not significantly different (**Figure 3B**; Tukey’s HSD; p = 0.76). Females reared in short-day conditions that were injected with *β-gal* dsRNA were in a clear diapause state, with an average egg follicle length of 53.3 ± 0.4 μm. However, the average egg follicle length of females reared in short-day conditions that were injected with *CpMRJP1* dsRNA was 70.7 ± 2.7 μm, and females were found to be in a mixture of diapause (70%), intermediate (25%), and nondiapause (5%) states. Overall, knocking down *CpMRJP1* significantly increased egg follicle length in short-day reared females (Tukey’s HSD; p < 0.001; 95% [CI = 10.4, 24.4]).

We also used a two-way ANOVA to determine whether photoperiod and knocking down *CpMRJP1* affected fat content (**Supplementary Figure S3B**). Our analyses revealed that dsRNA injection did not affect fat content (F_1, 26_ = 2.28, p = 0.143). However, photoperiod had a significant impact on fat content (F_1, 26_ = 4.47; p = 0.045), such that *β-gal* and *MRJP1* dsRNA-injected, short-day reared females had higher levels of fat than dsRNA-injected long-day reared females. This was largely driven by a non-significant trend where females reared in long-day conditions and injected with *β-gal* dsRNA had slightly less fat (6.45 ± 0.78%) than long day-reared mosquitoes injected with *CpMRJP1* dsRNA (10.97 ± 0.40%; Tukey’s HSD, p = 0.181), and short-day reared mosquitoes injected with *β-gal* dsRNA (Tukey’s HSD, p = 0.092) or *CpMRJP1* dsRNA (Tukey’s HSD, p = 0.072). Our 2-way ANOVA also confirmed that that there was not a significant interaction between photoperiod and dsRNA injection (F_1, 26_ = 2.12, p = 0.157).

We also investigated how injection with *MRJP1* dsRNA affected the lifespan of long and short day-reared females relative to *β-gal* dsRNA-injected controls in the absence of food (**Figure 4C**). A Cox proportional hazards model revealed that short photoperiods had a protective effect on female lifespan in the absence of food (**Supplementary Table S1**; HR = -1.31 ± 0.209; z = -6.28, p < 0.001), while knocking down *CpMRJP1* had a slight, but non-significant protective effect (HR = -0.307 ± 0.167; z = -1.83; p = 0.0668). The median survival time of long-day reared, *β-gal* dsRNA-injected females was six weeks while the median survival time of short-day reared, *β-gal* dsRNA-injected females was eleven weeks (Tukey’s HSD, z = -6.28, p < 0.001). The median survival time of long-day reared, *CpMRJP1* dsRNA-injected females was four weeks, while the median survival time of short-day reared, *CpMRJP1* dsRNA-injected females was nine weeks (Tukey’s HSD, z = -1.314, p < 0.001). While knocking down *CpMRJP1* did not significantly affect the likelihood of dying in females reared in long-day conditions relative to *β-gal* injected controls (Tukey’s HSD, z = -1.833, p = 0.258), dsRNA against *CpMRJP1* significantly extended the total lifespan in short-day conditions (**Figure 4C**; all *β-gal*-injected females were dead at 11 weeks; all *MRJP1-*dsRNA injected females were dead at 15 weeks; Log Rank Test of survival differences: X^2^_1_ = 5.9, p = 0.02).

## 4 Discussion

Our phylogenetic analyses show that CpMRJP1 forms a unique clade with MRJP’s in the paddled-beauty mosquito, *Sabethes cyaneus*, and *Ae. aegypti* (**Figure 1B**). Additionally, our results demonstrate that ingesting royal jelly reverses some, but not all, of the phenotypes associated with diapause in female mosquitoes. Specifically, female mosquitoes reared in long-day, diapause-averting conditions that consumed a diet including royal jelly enter a diapause-like state with small egg follicles (**Figure 2B**). In contrast, consuming a diet that includes royal jelly significantly reduced the lifespan of females reared in short-day, diapause-inducing conditions (**Figure 2C**). While consuming a diet containing royal jelly does not cause significant differences in fat content within long or short day-reared mosquitoes (**Supplementary Figure S1A**), it alters the metabolic profile of mosquitoes (**Figure 3**; **Supplementary Figure S2**; **Supplementary Table S1**). Specifically, long-day reared mosquitoes that consumed a diet containing royal jelly were metabolically similar to diapausing controls, and there were no significant differences in the relative abundance of 5/6 metabolites we identified between these two groups (**Figure 3B**; **Supplementary Table S2**). In contrast, short-day reared mosquitoes that consumed a diet containing royal jelly were metabolically similar to nondiapausing controls as there were no significant difference in any of the identified metabolites between long-day reared females that consumed sugar and short-day reared females that consumed diets with royal jelly (**Figure 3B**; **Supplementary Table S2**). As royal jelly is a complex mixture containing several proteins, carbohydrates, lipids and vitamins, it is currently not clear how royal jelly might be mediating these effects. However, it is likely that some of the traits we observed are induced through the action of MRJP1. This is because knocking down *CpMRJP1* significantly increased the egg follicle lengths of short-day reared females (**Figure 4B**) and allowed them to live significantly longer than *β-gal* dsRNA-injected controls in the absence of food (**Figure 4C**).

Transcripts encoding CpMRJP1, an ortholog of MRJP1 in *Apis mellifera* that is the most abundant protein in royal jelly, were upregulated in diapausing females (18). Another study demonstrated that consuming royal jelly increased the likelihood that alfalfa leaf cutting bees would enter diapause (25). Therefore, we hypothesized that consuming diets that include royal jelly, and thereby artificially increasing the levels of AmMRJP1 within mosquitoes, would induce diapause phenotypes in long-day reared mosquitoes. Although our current study did not show any significant differences in *CpMRJP1* mRNA abundance between diapausing and nondiapausing, sugar-fed mosquitoes (**Figure 2A**), we found that long day-reared females that consumed diets containing royal jelly had significantly smaller egg follicles (**Figure 2B**). This indicates that feeding female mosquitoes royal jelly in conditions that typically prevent diapause causes them to arrest reproductive development. However, in contrast to our results, consuming royal jelly promotes reproductive development in honey bee queens (15) and fruit flies (23). As other species also become more virile and fecund upon consuming royal jelly (22, 23), there must be a separate, unique pathway through which royal jelly acts in *Cx. pipiens* to confer reproductive arrest. We believe these effects are mediated through CpMRJP1 because knocking down this transcript stimulated reproductive development in mosquitoes reared in short day, diapause-inducing conditions (**Figure 4B**).

Reproductive arrest is not the only indicator of diapause, as diapausing females of *Cx. pipiens* also display an increase in fat content (37, 42, 59). We observed a trend where long-day reared females that consumed royal jelly had slightly higher fat content than females that consumed sucrose solution (**Supplementary Figure S1A**). However, due to high variation within our samples, the fat content was not significantly different between rearing conditions or food source. Typically, the increase in fat content is a consequence of the feeding habits of female mosquitoes; short-day reared females gorge on nectar that is rich in sugar (5, 6, 9) which they then convert into lipids (9). In contrast, nondiapausing mosquitoes do not accumulate substantial levels of fat (9, 37, 42). These differences are not likely not driven by lipid deposition into egg follicles as other researchers found that female mosquitoes of *Ae. aegypti* only deposit lipids into their egg follicles several hours after blood-feeding (60), and none of the mosquitoes in our study were given access to vertebrate blood. Although it is unclear why we did not observe significant increases in the fat content of diapausing females relative to nondiapausing sugar-fed controls, the absence of an effect of consuming diets that include royal jelly could be the result of nutritional differences in royal jelly (14), and/or because female mosquitoes in the royal jelly treatment may not have consumed as much food as the sugar-fed, control mosquitoes. Unfortunately, we did not measure how much food the mosquitoes consumed in our experiments. However, we conducted follow-up experiments demonstrating that long and short-day reared mosquitoes die within six days of adult emergence if they are provided access to only water. All our phenotypic measurements were collected from females that were seven-days old, indicating that they must have consumed at least some of their respective diets.

As royal jelly contains a high proportion of protein (13, 14), we chose to examine whether protein content changed between females that consumed diets that included royal jelly relative to those who fed on only sucrose (**Supplementary Figure S1B**). Consuming diets containing royal jelly did not affect the protein content of long or short-day reared mosquitoes, possibly because females in the royal jelly treatment may not have consumed as much food as those in the sucrose treatment as discussed above. However, we did find that females reared in long-day conditions have significantly greater protein content than those reared in short-day conditions. Early in diapause, females of *Cx. pipiens* produce fewer proteins than they do upon diapause termination (61). Furthermore, diapausing female mosquitoes are relatively inactive and take refuge in protected shelters (62), so they would not require as much protein to power their flight muscles.

In addition to determining how supplementing the diet of mosquitoes with royal jelly would affect seasonal phenotypes, we used RNAi to elucidate the functional role of CpMRJP1 in diapausing females of *Cx. pipiens*. We were able to successfully knock-down the relative abundance of *CpMRJP1* transcripts in both long and short-day reared *Cx. pipiens* (**Figure 4A**). However, knocking down *CpMRJP1* only induced phenotypic effects in short-day reared females, where it significantly increased the egg follicle length of 7-day old mosquitoes such that approximately 30% of the females sampled were categorized as being in an intermediate or nondiapause state (**Figure 4B**). Therefore, knocking down *CpMRJP1* causes short-day reared females to avert diapause, and again suggests that the gene encoding CpMRJP1 plays a critical role in arresting egg follicle development during diapause induction in females of *Cx. pipiens*.

Knocking down *CpMRJP1* did not lead to a significant change in fat content of females reared in short-day conditions (**Supplementary Figure S3B**), although, we observed a trend in which the long day-reared females that were injected with *CpMRJP1* dsRNA had a slight, but non-significant, higher level of fat compared to *β-gal* dsRNA-injected controls. This is surprising, seeing as *CpMRJP1* mRNA is upregulated in diapausing mosquitoes that acquire high levels of fat (18), but we found that the fat content slightly increased when *CpMRJP1* was knocked down with RNAi. Overall, the female mosquitoes that were injected with *CpMRJP1* or *β-gal* dsRNA had lower levels of fat (**Supplementary Figure S3B**) than the female mosquitoes that were not injected and allowed to consume sugar water in the initial dietary experiment (**Supplementary Figure S1A**). Therefore, we conclude that injecting mosquitoes likely caused some minor injuries that impaired their ability to consume the sucrose source that was within their cage. These results, combined with the effects of consuming royal jelly, suggest that CpMRJP1 may not be directly involved in accumulating fat during diapause and rather that this protein regulates reproductive development and/or starvation resistance.

Consuming diets that contained royal jelly for one week prior to food removal significantly reduced the median survival time in the absence of food for both long and short-day reared mosquitoes, relative to mosquitoes that consumed 10% sucrose for one week before food removal (**Figure 2C**). The decrease in survival was most dramatic and pronounced in mosquitoes reared in short-day, diapause-inducing conditions where consuming diets that included royal jelly reduced the median lifespan by 12 weeks. This is a surprising result, seeing as royal jelly increases the lifespan of honey bees and fruit flies (14, 23). Additionally, in short-day conditions, knocking down *MRJP1* with RNAi increased lifespan (**Figure 4C**). Although *CpMRJP1* dsRNA-injected mosquitoes initially died sooner and had a lower median survival time than *β-gal* dsRNA injected controls, knocking down *CpMRJP1* in short day-reared mosquitoes extended their lifespan by four weeks. Taken together, our data suggest that a factor within royal jelly, possibly MRJP1, may reduce the starvation resistance of diapausing mosquitoes.

We found six metabolites that were significantly differentially abundant between diapausing and nondiapausing mosquitoes as well as those that had consumed royal jelly (**Figure 3B**). It is obvious that consuming royal jelly caused largescale changes in whole mosquito metabolomes that partially reversed the seasonal phenotypes of the mosquitoes (**Figure 3A**), and these results were further supported by artificial neural network predictions. In addition, mosquitoes reared in short-day, diapause-inducing conditions that consumed diets that included royal jelly had a metabolic profile that was similar to nondiapausing mosquitoes, such that both long-day reared, sugar-fed controls and short-day reared, royal jelly-fed mosquitoes had significantly lower levels of asparagine, choline, and pimelic acid as well as higher levels of glycogen, and histidine (**Figure 3B**). These metabolic results are consistent with the phenotypes we observed, where ingesting diets that contained royal jelly both induced diapause phenotypes in long-day reared mosquitoes (e.g., reduced egg follicle length) and caused short-day reared females to exhibit nondiapause phenotypes (e.g., reduced starvation tolerance).

A pathway enrichment analysis (**Figure 3C**) revealed several metabolic pathways that were affected by day length and/or royal jelly consumption. One of the affected pathways is the biotin metabolism pathway, with its metabolite pimelic acid being upregulated during diapause and in long day-reared mosquitoes that consumed royal jelly (**Figure 3B**). Pimelic acid is a precursor of biotin (vitamin B7) that can be made during fatty acid synthesis, specifically, it has been linked to the FabF enzyme in the bacteria *Bacilus subtilis* (63). While FabF is also part of fatty acid synthesis in *Cx. pipiens*, and fatty acid synthesis is upregulated in diapause (5), no pimelic acid synthesis has been previously reported for this organism, and further experiments would be required to investigate this possibility. Pimelic acid is also a metabolite in mosquito microbiomes (64). Therefore, microbial contributions may explain the observed change. Supplementing the diets of honey bee with pimelic acid has been shown to decrease stress responses and increasing survival times (65). Decreased levels of pimelic acid could thus partially explain the decreased life expectancy of short day-reared mosquitoes that consumed royal jelly. The observed increase in pimelic acid in diapause-inducing conditions also indicates a potential microbial contribution to mosquito diapause.

Our analyses also show that the starch and sucrose metabolism pathway was similarly affected (**Figure 3C**), with glycogen being less abundant in diapause and in long-day reared mosquitoes that consumed diets that included royal jelly (**Figure 2B**). A previous study reports metabolic flux of dietary glucose toward glycogen in diapausing *Cx. pipiens* (66). However, that study did not analyze glycogen in nondiapausing animals and thus cannot be directly compared to our data. In accordance with our results, Zhou and Meisfeld (67) found that glycogen decreases during the first weeks of diapause in *Cx. pipiens* as compared to nondiapausing mosquitoes, with a simultaneous increase in body fat. Our findings suggest that consuming diets that contain royal jelly significantly reduced the diapause-associated catabolism of glycogen in short-day reared mosquitoes.

The alanine, aspartate and glutamate metabolism (AAGM) pathway was also significantly affected. One metabolite in this pathway, L-alanine, was less abundant in diapausing mosquitoes and females reared in long-day conditions that consumed royal jelly (**Figure 3B**). Alanine can, via pyruvate and the glycolysis/gluconeogenesis pathway, feed into starch and sucrose metabolism. Therefore, our finding that the abundance of L-alanine decreased diapausing mosquitoes is consistent with the observed drop in glycogen as discussed above. Alanine is also downregulated in diapausing *N. vitripennis* (28), but is more abundant in diapausing flesh flies, where it likely functions as a cryoprotectant (27). We also found that asparagine, another metabolite of the AAGM pathway, was significantly upregulated in long-day reared mosquitoes that consumed royal jelly (**Figure 3B**). Asparagine was upregulated in diapausing pupae of the moth *Antheraea pernyi* (68) but was less abundant in diapausing larvae of the parasitoid *N. vitripennis* (28). Together with the downregulation of L-alanine, these results suggest that L-aspartate is preferably converted into L-asparagine rather than L-alanine in diapausing *Cx. pipiens* to meet their differing metabolic needs.

Glycerophospholipid metabolism also changed across photoperiods and dietary treatments (**Figure 3C**), such that choline was upregulated in diapausing mosquitoes and in long-day reared mosquitoes that consumed diets including royal jelly (**Figure 3B**). As choline is a precursor of phosphatidylcholine, the observed differences suggest that there are differences in phospholipid synthesis during diapause. This could indicate that diapausing insects are catabolizing phospholipids to generate precursors for triglycerides, which would be consistent with previous studies that have shown that triglyceride stores increase during diapause in *Cx. pipiens* (5, 9) and other insects (reviewed in 69). However, this finding could also indicate that diapausing insects are synthesizing more and/or different phospholipids. Previous studies have demonstrated that diapausing insects increase the concentration of unsaturated fatty acids within cell membranes to increase membrane fluidity and enhance cold tolerance (69–71). Future studies are necessary to distinguish whether phospholipid catabolism and/or anabolism are occurring within diapausing *Cx. pipiens*.

Both photoperiod and diet affected histidine metabolism (**Figure 3C**), such that histidine was significantly downregulated in diapausing *Cx. pipiens* and long-day reared mosquitoes that consumed diets containing royal jelly (**Figure 3B**). Although histidine is upregulated in diapausing *N. vitripennis* (28), histidine is less abundant in diapause-destined larvae of the cotton bollworm, *Heliocoverpa armigera* (72). In pre-diapausing *H. armigera*, down-regulating histidine likely leads to lower levels of its byproduct histamine, an inhibitory neurotransmitter, that may alter the photoperiodic responses necessary for diapause induction (72).

Notably, royal jelly contains multiple proteins, lipids, vitamins, and carbohydrates (13), and any of these components could have directly or indirectly altered mosquito reproductive development, starvation resistance and metabolic profiles. Our RNAi experiments support the findings of our feeding experiments; specifically, consuming royal jelly (thereby artificially increasing the abundance of AmMRJP1 within mosquitoes) decreased egg follicle length in long-day reared mosquitoes, whereas knocking down *CpMRJP1* increased egg follicle length short-day reared females. Taken together, this suggests that higher levels of MRJP1 promotes reproductive arrest. However, at this time we still do not know precisely where and when *CpMRJP1* is being expressed or how it might be mediating these effects. It is also unclear if consuming AmMRJP1 on its own or other components in royal jelly promotes the metabolomic switch that we observed. Therefore, future studies are necessary to characterize the mechanism by which MRJP1 promotes reproductive arrest in *Cx. pipiens* and to delineate the effects of MRJP1 from other components in royal jelly.

## 5 Conclusions

This study demonstrates that consuming diets that include royal jelly has opposing effects on phenotypes associated with diapause in *Cx. pipiens*. In long-day reared mosquitoes, these include suppressing reproductive development and causing the metabolomic profile to resemble that of diapausing females. In contrast, in short-day reared females, consuming diets that include royal jelly reduces starvation resistance and shifts the metabolomic profile to be more similar to long-day reared mosquitoes. As royal jelly is a complex mixture of multiple proteins, sugars, lipids, vitamins and other substances (13), it is currently unclear how consuming royal jelly switches seasonal phenotypes in *Cx. pipiens*; but we can conclude that this effect on diapause was mediated at least in part by MRJP1. This is because knocking down *CpMRJP1* caused females that were reared in short-day, diapause-inducing conditions to avert diapause and develop significantly larger egg follicles and to live significantly longer than *β-gal* dsRNA injected controls. As diapausing females of *Cx. pipiens* do not bite humans and other animals (6), they do not transmit debilitating diseases (62). Future work should investigate whether it would be possible to develop control measures that use royal jelly to induce diapause in female mosquitoes during the long days of summer to reduce disease transmission. Additionally, future work should be done to elucidate whether AmMRJP1 alone or other components of royal jelly induce reproductive arrest, cause largescale metabolic shifts and alter mosquito starvation resistance. Such studies will not only uncover the underpinnings of the interesting results we observed in this study, but may also lead to exciting insights on the molecular regulation of seasonal responses in other insects and animals.

## Data availability statement

The original contributions presented in the study are included in the article/**Supplementary Materials**, further inquiries can be directed to the corresponding author/s.

## Ethics statement

The manuscript presents research on animals that do not require ethical approval for their study.

## Author contributions

OB: Conceptualization, Data curation, Formal Analysis, Investigation, Methodology, Writing – original draft, Writing – review & editing. AA: Investigation, Methodology, Writing – review & editing. MK: Data curation, Formal Analysis, Investigation, Methodology, Software, Supervision, Visualization, Writing – review & editing. XW: Investigation, Methodology, Software, Visualization, Writing – review & editing. CS: Investigation, Methodology, Software, Supervision, Visualization, Writing – review & editing. MM: Conceptualization, Formal Analysis, Funding acquisition, Investigation, Project administration, Resources, Supervision, Validation, Visualization, Writing – original draft, Writing – review & editing.
