# Supplementary material for: Consuming royal jelly alters several phenotypes associated with overwintering dormancy in mosquitoes
Source: Front Insect Sci. 2024 Jun 7;4:1358619. doi: 10.3389/finsc.2024.1358619 (PMC11190361; doi:10.3389/finsc.2024.1358619)
Supplement: Supplementary file 1 [file DataSheet_1.pdf]

Supplementary Material

Consuming royal jelly alters several phenotypes associated with overwintering dormancy in mosquitoes

Olivia E. Bianco, Aisha Abdi, Matthias S. Klein, Xueyan Wei, Cheolho Sim, Megan E. Meuti*

*** Correspondence:** Corresponding Author: meuti.1@osu.edu

1 Supplementary Figures and Tables

1.1 Supplementary Figures

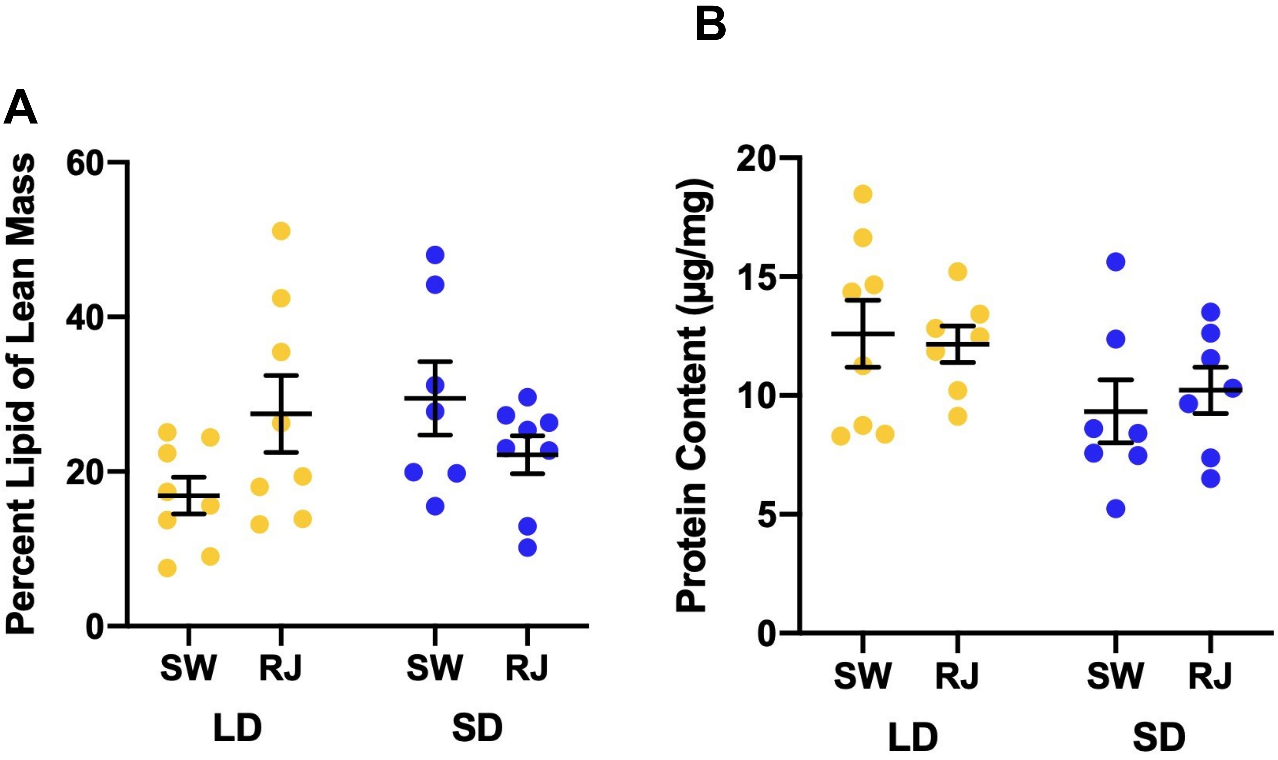

Fig. S1: Consuming royal jelly (RJ) did not significantly impact whole body fat (A) or protein (B) content in female mosquitoes relative to females that consumed sugar water (SW) in females that were reared in long day, diapause-averting conditions (LD) or short day, diapause-inducing conditions (SD). A. Fat content was measured using a modified vanillin assay. B. Protein levels were measured using a Bradford assay. Although dietary treatments did not affect the protein content, LD mosquitoes had significantly more protein than all SD mosquitoes. Significant difference denoted by * (p = 0.03).

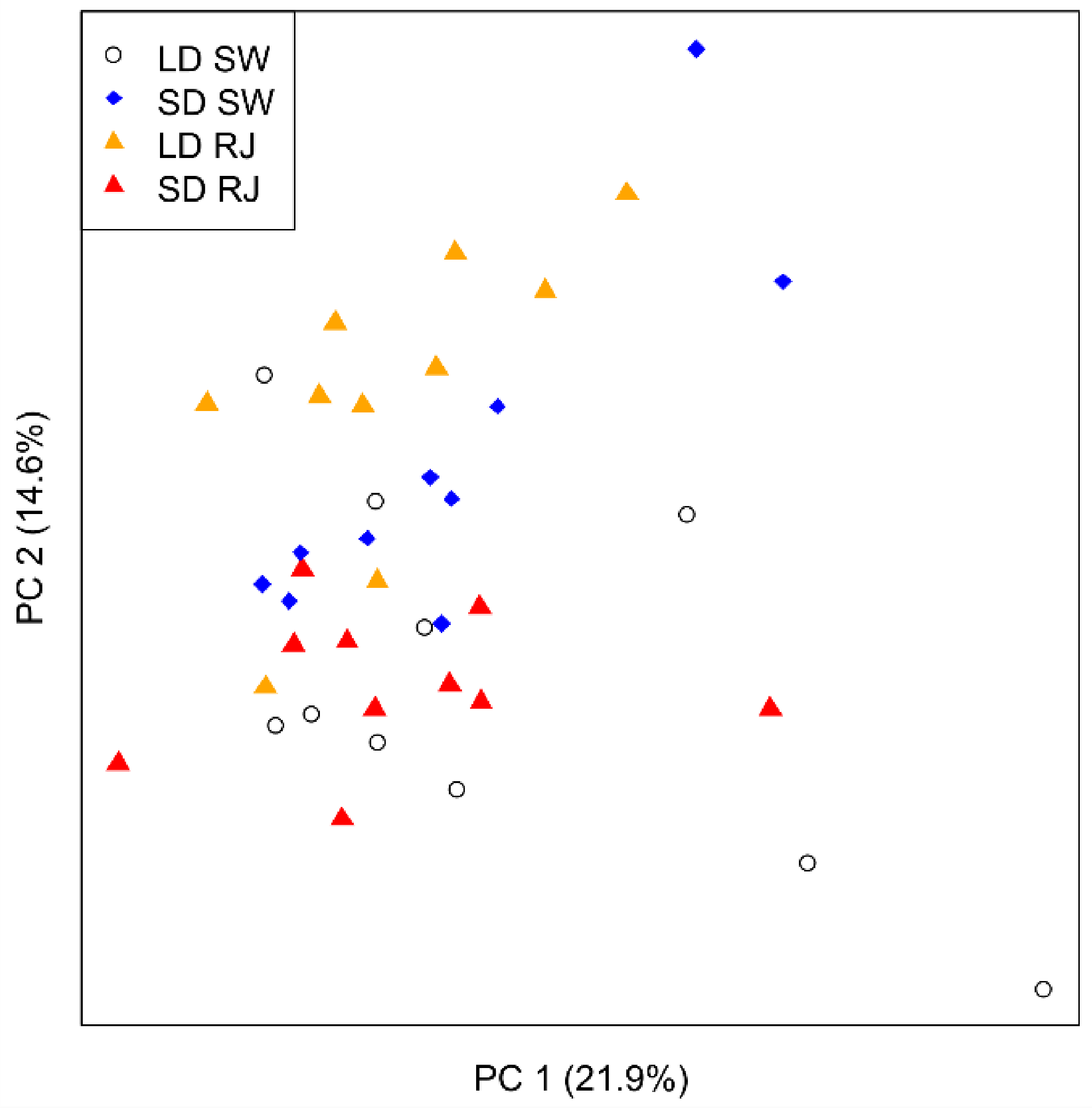

Fig. S2: Principal Component Analysis (PCA) plot of 1D NMR metabolomics data illustrating that dietary and rearing conditions have a significant effect on mosquitoes' metabolic profiles.

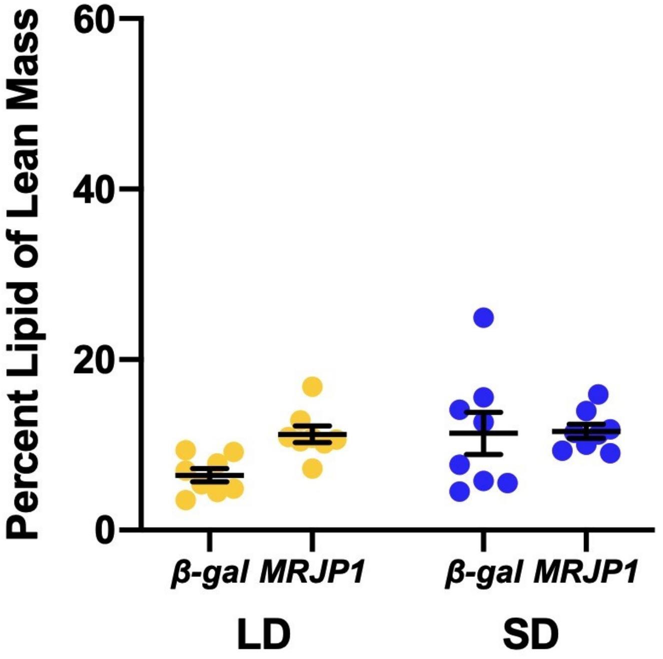

Fig. S3: Treatment with dsRNA for *β-gal* or *MRJP1* did not significantly affect fat content of female mosquitoes reared in long day (LD) or short day (SD) conditions. A.) A second, independent round of injections with *MRJP1* and *β-gal* dsRNA failed to significantly change the level of *MRJP1* mRNA abundance. RNA was isolated from mosquitoes 2 days after dsRNA injections and measured with qRT-PCR. B.) Lipid levels were measured with a modified Vanillin assay.

1.2 Supplementary Tables

Table S1: List of spectral regions (bins) exhibiting significant correlations to dietary treatment (sugar water control = 0, royal jelly = 1), photoperiodic conditions (long days/nondiapausing = 0, short days/diapausing = 1), and/or the interaction term. Unambiguously identified metabolites are labeled. Coefficients indicate whether the metabolite is positively or negatively correlated to the respective factor (positive/negative coefficients). Overall, results demonstrate that for most metabolites, consuming royal jelly reverses the changes seen in diapause.

	Significant				Coefficients		
Bin borders (chemical shift δ) [ppm]	Royal Jelly	Diapause	Interaction Between Royal Jelly & Diapause	Metabolite identity	Royal Jelly	Diapause	Interaction Between Royal Jelly & Diapause
0.671,0.668			*		1.09	1.04	-1.99
0.695,0.692			*		1.4	0.8	-2.26
0.743,0.74			*		-1.31	-0.87	1.97
1.025,1.022	*				1.25	-0.12	-0.36
1.028,1.025	*				1.45	0.64	-0.73
1.142,1.139			*		-0.8	-1.17	2.1
1.148,1.145			*		-1	-1.44	2.03
1.151,1.148			*		-1.15	-1.47	2.08
1.181,1.178			*		-1.25	-1.24	2.07
1.187,1.184			*		-1.28	-1.21	2.16
1.19,1.187			*		-1.23	-0.95	2.02

	Significant				Coefficients		
**Bin borders (chemical shift δ) [ppm]**	**Royal Jelly**	**Diapause**	**Interaction Between Royal Jelly & Diapause**	**Metabolite identity**	**Royal Jelly**	**Diapause**	**Interaction Between Royal Jelly & Diapause**
1.196,1.193			*		-1.31	-1.12	2.17
1.199,1.196			*		-1.36	-1.08	2.27
1.208,1.205			*		-1.04	-0.96	2.22
1.22,1.217			*		-0.69	-0.95	2.07
1.331,1.328			*	pimelic acid	1.2	0.84	-2.2
1.34,1.337			*	pimelic acid	1.07	0.98	-2.09
1.343,1.34			*	pimelic acid	0.97	1.03	-2.08
1.346,1.343			*	pimelic acid	1.04	1.13	-2.18
1.349,1.346			*	pimelic acid	1.04	1.14	-2.21
1.352,1.349			*	pimelic acid	0.93	1.09	-2.09
1.436,1.433			*		1.06	1.65	-2.01
1.439,1.436			*		0.81	1.47	-1.97
1.481,1.478		*		L-Alanine	-0.86	-1.93	1.52

	**Significant**				**Coefficients**		
**Bin borders (chemical shift δ) [ppm]**	**Royal Jelly**	**Diapause**	**Interaction Between Royal Jelly & Diapause**	**Metabolite identity**	**Royal Jelly**	**Diapause**	**Interaction Between Royal Jelly & Diapause**
1.49,1.487		*		L-Alanine	-0.76	-1.87	1.36
1.493,1.49		*		L-Alanine	-1.04	-1.79	1.46
1.538,1.535			*	pimelic acid	1.07	1.01	-2.03
1.541,1.538			*	pimelic acid	1.11	1.03	-2.14
1.544,1.541			*	pimelic acid	1.05	1.08	-2.14
1.547,1.544			*	pimelic acid	1.03	1.14	-2.21
1.55,1.547			*	pimelic acid	1.12	1.05	-2.25
1.559,1.556			*	pimelic acid	1	0.95	-2.05
1.562,1.559			*	pimelic acid	0.96	0.86	-2
1.565,1.562			*	pimelic acid	0.98	1.01	-2.02
1.7495,1.7055		*			0.29	1.57	-1.12
1.898,1.895		*			-0.1	1.57	-1.17

	Significant				Coefficients		
Bin borders (chemical shift δ) [ppm]	Royal Jelly	Diapause	Interaction Between Royal Jelly & Diapause	Metabolite identity	Royal Jelly	Diapause	Interaction Between Royal Jelly & Diapause
2.171,2.168			*	pimelic acid	1.08	1.11	-2.17
2.174,2.171			*	pimelic acid	0.95	0.97	-2
2.177,2.174			*	pimelic acid	0.97	0.99	-2.03
2.18,2.177			*	pimelic acid	1.03	1.1	-2.07
2.381,2.378			*		1.34	0.66	-1.97
2.387,2.384			*		1.31	0.29	-1.86
2.396,2.393			*		1.41	0.68	-1.98
2.531,2.528			*		1.52	0.34	-2.02
2.546,2.543			*		1.25	0.54	-2.06
2.573,2.57		*			1.33	-0.08	-1.58
2.609,2.606			*		1.37	0.15	-1.78
2.612,2.609			*		1.44	0.14	-1.76

	Significant				Coefficients		
Bin borders (chemical shift δ) [ppm]	Royal Jelly	Diapause	Interaction Between Royal Jelly & Diapause	Metabolite identity	Royal Jelly	Diapause	Interaction Between Royal Jelly & Diapause
2.651,2.648		*			0.82	-0.49	-0.95
2.69,2.687			*		1.17	0.31	-1.89
2.771,2.77		*			0.83	-0.37	-1.18
2.83,2.828			*		1.47	0.35	-2.11
2.862,2.835			*	Asparagine	1.52	0.18	-1.95
2.903,2.9			*		1.31	0.11	-1.8
2.909,2.906		*	*		1.43	0.2	-2.18
2.924,2.921		*			0.46	-0.67	-0.79
2.927,2.924		*			0.76	-0.35	-1.57
2.93,2.927			*		1.14	0.08	-1.87
2.9505,2.93			*	Asparagine	1.46	0.41	-2.27

	Significant				Coefficients		
Bin borders (chemical shift δ) [ppm]	Royal Jelly	Diapause	Interaction Between Royal Jelly & Diapause	Metabolite identity	Royal Jelly	Diapause	Interaction Between Royal Jelly & Diapause
2.971,2.9505			*	Asparagine	1.3	0.27	-1.93
3.15,3.1		*		Histidine	-1.19	-1.68	1.57
3.158,3.155			*		1.14	0.54	-1.99
3.203,3.2			*	Choline	1.29	1.04	-2.38
3.206,3.203			*	Choline	1.4	1.57	-2.92
3.209,3.206			*	Choline	1.59	1.64	-3.15
3.212,3.209			*	Choline	1.16	1.03	-2.22
3.224,3.221			*		1.41	1.66	-3.2
3.227,3.224			*		1.74	1.78	-3.37
3.233,3.23			*		0.41	0.57	-2.09
3.242,3.239	*			Histidine	-1.01	0.25	0.06
3.29,3.287		*			-0.57	-1.4	0.63
3.296,3.293		*			-0.69	-1.51	0.82

	Significant				Coefficients		
Bin borders (chemical shift δ) [ppm]	Royal Jelly	Diapause	Interaction Between Royal Jelly & Diapause	Metabolite identity	Royal Jelly	Diapause	Interaction Between Royal Jelly & Diapause
4.163,4.16			*		0.53	1.53	-1.92
4.166,4.163			*		0.98	1.83	-2.63
4.277,4.274			*		-0.8	-0.69	2.12
4.28,4.277			*		-0.88	-0.86	1.98
4.283,4.28			*		-1.13	-0.88	2.41
4.286,4.283			*		-1.43	-1.27	2.83
4.289,4.286			*		-1	-0.93	1.99
4.307,4.304			*		0.73	1.28	-1.96
4.319,4.316			*		1.11	1.59	-2.58
4.322,4.319			*		1.06	1.24	-2.35
4.325,4.322			*		0.96	1.17	-2.63
4.328,4.325			*		0.86	1.03	-2.64
4.331,4.328			*		0.84	1.05	-2.63

	Significant				Coefficients		
Bin borders (chemical shift δ) [ppm]	Royal Jelly	Diapause	Interaction Between Royal Jelly & Diapause	Metabolite identity	Royal Jelly	Diapause	Interaction Between Royal Jelly & Diapause
4.334,4.331			*		0.8	1.1	-2.57
4.337,4.334			*		0.86	1.12	-2.65
4.34,4.337			*		0.9	1.23	-2.64
4.343,4.34			*		0.81	1.3	-2.45
4.346,4.343			*		0.82	1.37	-2.2
4.352,4.349			*		1.13	1.57	-2.38
4.358,4.355			*		0.9	1.5	-2.14
4.436,4.433			*		-1.42	-1.32	2.14
4.442,4.439			*		-1.59	-1.58	2.48
4.448,4.445			*		-1.53	-1.74	2.45
4.955,4.952			*		1.1	1.35	-2.35
4.958,4.955			*		0.96	1.31	-2.33
4.961,4.958			*		0.85	1.01	-2.09

	Significant				Coefficients		
Bin borders (chemical shift δ) [ppm]	Royal Jelly	Diapause	Interaction Between Royal Jelly & Diapause	Metabolite identity	Royal Jelly	Diapause	Interaction Between Royal Jelly & Diapause
5.177,5.174		*			-0.45	1.27	-0.62
5.249,5.246			*		0.75	0.99	-2.01
5.324,5.321			*		-1.17	-1.27	2.54
5.339,5.336			*		-1.38	-1.26	2.03
5.351,5.348			*		-1.4	-1.07	1.95
5.402,5.399			*	Glycogen	-1.62	-1.09	2.05
5.405,5.402			*	Glycogen	-1.67	-1.04	2.04
5.408,5.405			*	Glycogen	-1.59	-1.13	2.04
5.411,5.408			*	Glycogen	-1.48	-1.1	1.94
5.435,5.432			*	Glycogen	-1.23	-0.76	1.98
5.513,5.51			*		-1.75	-1.47	1.85
5.807,5.804			*		0.97	1.06	-2.05

	Significant				Coefficients		
Bin borders (chemical shift δ) [ppm]	Royal Jelly	Diapause	Interaction Between Royal Jelly & Diapause	Metabolite identity	Royal Jelly	Diapause	Interaction Between Royal Jelly & Diapause
5.81,5.807			*		1.04	1.24	-2.19
5.828,5.825			*		1.05	1.08	-2.15
5.858,5.855		*	*		0.43	2	-1.76
5.87,5.867		*			0.41	1.87	-1.61
6.113,6.097			*		-1.29	-1.26	2.1
6.151,6.1482	*				-1.2	-0.02	0.22
6.65,6.647			*		1.07	1.01	-2.13
6.659,6.656			*		0.99	1.12	-2.08
6.668,6.665			*		1.18	1.06	-2.17
6.713,6.71			*		-0.49	-0.36	1.86
6.836,6.833			*		-1.17	-0.95	2.03
7.074,7.04			*	Histidine	-1.68	-1.48	2.53

	Significant				Coefficients		
Bin borders (chemical shift δ) [ppm]	Royal Jelly	Diapause	Interaction Between Royal Jelly & Diapause	Metabolite identity	Royal Jelly	Diapause	Interaction Between Royal Jelly & Diapause
7.703,7.674			*		-1.58	-1.05	2.32
7.81,7.735			*	Histidine	-1.54	-1.02	2.23
8.219,8.216		*			0.72	1.63	-1.36
8.223,8.222		*			0.63	1.69	-1.26
8.353,8.334			*		-1.23	-1.23	1.97
9.011,9.008		*	*		0.72	2.04	-1.53
9.02,9.017		*			0.14	1.71	-1.36
9.101,9.098		*	*		0.51	2.01	-1.77
9.107,9.104		*	*		0.68	1.88	-1.86
9.35,9.347		*	*		0.62	1.93	-1.96
9.353,9.35		*	*		0.44	1.9	-1.78

Table S2: Prediction table of the artificial neural network (ANN) cross validation results for the training set (sugar water group).

		True	
		**Long day**	**Short day**
**Predicted**	**Long day**	80%	0%
	**Short day**	20%	100%

Table S3: Prediction table of the artificial neural network (ANN) prediction results for the test set (royal jelly group). Please note, the ANN was trained exclusively on the sugar water group samples.

		True	
		**Long day**	**Short day**
**Predicted**	**Long day**	38.5%	78.5%
	**Short day**	61.5%	21.5%
